# Determinants of Dropout From a Virtual Agent–Based App for Insomnia Management in a Self-Selected Sample of Users With Insomnia Symptoms: Longitudinal Study

**DOI:** 10.2196/51022

**Published:** 2025-01-15

**Authors:** María Montserrat Sanchez Ortuño, Florian Pecune, Julien Coelho, Jean Arthur Micoulaud-Franchi, Nathalie Salles, Marc Auriacombe, Fuschia Serre, Yannick Levavasseur, Etienne De Sevin, Patricia Sagaspe, Pierre Philip

**Affiliations:** 1 Departamento de Enfermería, Universidad de Murcia, Murcia, 30120, Spain; 2 Laboratoire SANPSY, CNRS, UMR 6033, Université de Bordeaux-Centre Hospitalier Universitaire Pellegrin de Bordeaux, Bordeaux, France; 3 Laboratoire SANPSY, CNRS, UMR 6033, Université de Bordeaux-Pôle Interétablissement d’Addictologie, Bordeaux, France

**Keywords:** insomnia, digital behavioral therapy, mobile health, dropout, virtual agent–based app, virtual agent, user, digital intervention, smartphone, mental health, implementation, cognitive behavioral therapy, CBT

## Abstract

**Background:**

Fully automated digital interventions delivered via smartphone apps have proven efficacious for a wide variety of mental health outcomes. An important aspect is that they are accessible at a low cost, thereby increasing their potential public impact and reducing disparities. However, a major challenge to their successful implementation is the phenomenon of users dropping out early.

**Objective:**

The purpose of this study was to pinpoint the factors influencing early dropout in a sample of self-selected users of a virtual agent (VA)–based behavioral intervention for managing insomnia, named KANOPEE, which is freely available in France.

**Methods:**

From January 2021 to December 2022, of the 9657 individuals, aged 18 years or older, who downloaded and completed the KANOPEE screening interview and had either subclinical or clinical insomnia symptoms, 4295 (44.5%) dropped out (ie, did not return to the app to continue filling in subsequent assessments). The primary outcome was a binary variable: having dropped out after completing the screening assessment (early dropout) or having completed all the treatment phases (n=551). Multivariable logistic regression analysis was used to identify predictors of dropout among a set of sociodemographic, clinical, and sleep diary variables, and users’ perceptions of the treatment program, collected during the screening interview.

**Results:**

The users’ mean age was 47.95 (SD 15.21) years. Of those who dropped out early and those who completed the treatment, 65.1% (3153/4846) were women and 34.9% (1693/4846) were men. Younger age (adjusted odds ratio [AOR] 0.98, 95% CI 0.97‐0.99), lower education level (compared to middle school; high school: AOR 0.56, 95% CI 0.35‐0.90; bachelor’s degree: AOR 0.35, 95% CI 0.23‐0.52; master’s degree or higher: AOR 0.35, 95% CI 0.22‐0.55), poorer nocturnal sleep (sleep efficiency: AOR 0.64, 95% CI 0.42‐0.96; number of nocturnal awakenings: AOR 1.13, 95% CI 1.04‐1.23), and more severe depression symptoms (AOR 1.12, 95% CI 1.04‐1.21) were significant predictors of dropping out. When measures of perceptions of the app were included in the model, perceived benevolence and credibility of the VA decreased the odds of dropout (AOR 0.91, 95% CI 0.85‐0.97).

**Conclusions:**

As in traditional face-to-face cognitive behavioral therapy for insomnia, the presence of significant depression symptoms plays an important role in treatment dropout. This variable represents an important target to address to increase early engagement with fully automated insomnia management programs. Furthermore, our results support the contention that a VA can provide relevant user stimulation that will eventually pay out in terms of user engagement.

## Introduction

Chronic insomnia is a major global health concern owing to its high prevalence and its negative impact on mental and physical health [1,2]. The usual approach to treat chronic insomnia includes the prescription of medication, such as nonbenzodiazepine hypnotics [3-5]. Nonpharmacological approaches, such as cognitive behavioral therapy for insomnia (CBT-I), whose goal is to restructure a person’s sleeping behavior, is an evidence-based psychotherapy that has been shown to treat this condition effectively. CBT-I is considered as the first-line treatment option [5]. However, in practice, few people receive CBT-I. Overall, traditional CBT-I is perceived as complex to administer (ie, requiring trained therapists) and time consuming, giving rise to a treatment gap in insomnia care [6,7].

Digital interventions, which deliver therapeutic components via a web browser or smartphone app, have been proposed as a scalable, cost-effective way to meet the demand for CBT-I and address the important challenges associated with accessing traditional face-to-face CBT-I [8-10]. A considerable body of evidence supports the efficacy of fully automated (ie, requiring no human support) digital CBT-I renditions for the management of insomnia [11-14]. Although these fully automated digital CBT-I programs can display some differences (eg, number of weeks required to complete the treatment), generally they all ask the user to track their sleep daily by completing a sleep diary. They also incorporate algorithms to provide personalized feedback and a treatment program tailored to the user that usually comprises sleep hygiene practices and evidence-based behavioral recommendations known to improve chronic insomnia symptoms.

KANOPEE is a smartphone app developed in France that is designed to monitor and help manage insomnia complaints using components of CBT-I, such as sleep hygiene and stimulus control recommendations [15,16]. The app includes an animated character able to engage in face-to-face dialogue through verbal and nonverbal behavior, known in the literature as a virtual agent (VA) [17-19]. The program is designed to be completed in 17 days. It starts with a screening interview, followed by the completion of sleep diaries for a week. After this, the VA conducts another interview and sleep recommendations are provided. The individual is requested to enact these recommendations and continue completing sleep diaries for 10 additional days. At the end of this period, users undergo the posttreatment interview.

Although KANOPEE’s clinical efficacy has not been tested yet in randomized clinical trials, it has been preliminarily tested in real-world scenarios [10,20]. These studies have shown encouraging positive effects in insomnia and related symptoms in sizable samples of self-selected individuals in France [21-24]. Nonetheless, scrutiny of the real-world data collected so far shows that a substantial proportion of users stop using the app before completing the full treatment protocol. This issue is not exclusive to KANOPEE: high attrition per dropout rates are ubiquitous problems in fully automated digital health interventions [25,26]. Indeed, it has been acknowledged that a central challenge to the overall efficacy and broadly scalable implementation of digital interventions is the phenomenon of users dropping out early [27]. A growing body of research aims to address the problem of dropout by examining factors that might predict it. The premise of this line of research is that if users who are at a high risk of dropping out can be identified, researchers and intervention developers can supplement or modify the intervention with the goal of improving retention [28]. The main types of variables that have been used to predict dropout have included self-reported baseline data, such as demographics or symptom severity [29], or objective measures of intervention engagement, such as number of loggings and proportion of content completed, among others [28,30].

When exploring the predictors of dropout, analyzing at what point dropout occurs is also of importance. Many studies only report a final figure about the prevalence of dropout during the entire intervention. While in most digital health interventions there is a continuous dropout over time, it has been suggested that the majority of users tend to drop out after completing the first one or two modules [27], a phenomenon known as early dropout. Arguably, an important gap in this literature is to identify if there are different variables associated with dropout at different points through the treatment process [31]. Indeed, while baseline variables and initial perceptions of treatment may certainly be valuable to understand early dropout, they may not be as relevant when analyzing dropout once the user has been interacting with the app for several days or weeks. Therefore, further research is needed to help understand the dropout phenomenon at different time points [32].

As a first step to develop a refined version of the KANOPEE app, our overarching goal in this study was to identify users’ baseline characteristics and users’ perceptions of the app and of the VA that may predict early dropout. Furthermore, since KANOPEE could be used in a preventative manner (in users with subclinical insomnia symptoms) or by users with more severe insomnia symptoms, we explored whether predictors of early dropout differed between 2 profiles of users who could potentially benefit from the intervention: users with clinical insomnia symptoms and those with subclinical insomnia symptoms.

## Methods

### Study Design

We employed a case series study of a self-selected sample of individuals completing a free, fully automated insomnia management program named KANOPEE, available to the general population in France, during the period January 2021 to December 2022.

### Participants

Inclusion criteria were as follows: (1) age >17 years, (2) completion of the first screening phase (phase 1), and (3) the presence of insomnia symptoms, either clinical or subthreshold, at phase 1.

### Ethical Considerations

This study was approved by the Institutional Review Board of the University of Bordeaux, France (2021-A02606-35 CPP Est II). Before providing any personal or health-related data on the KANOPEE app, users had to sign the app’s digital consent form. Therefore, informed consent was obtained from users before any data collection. Data provided by users were anonymized, and participants did not receive any financial compensation for using the app. We obtained agreement with respect to the General Data Protection Regulations of the French authorities (the National Commission on Informatics and Liberty).

### Intervention Overview

**Figure 1.**
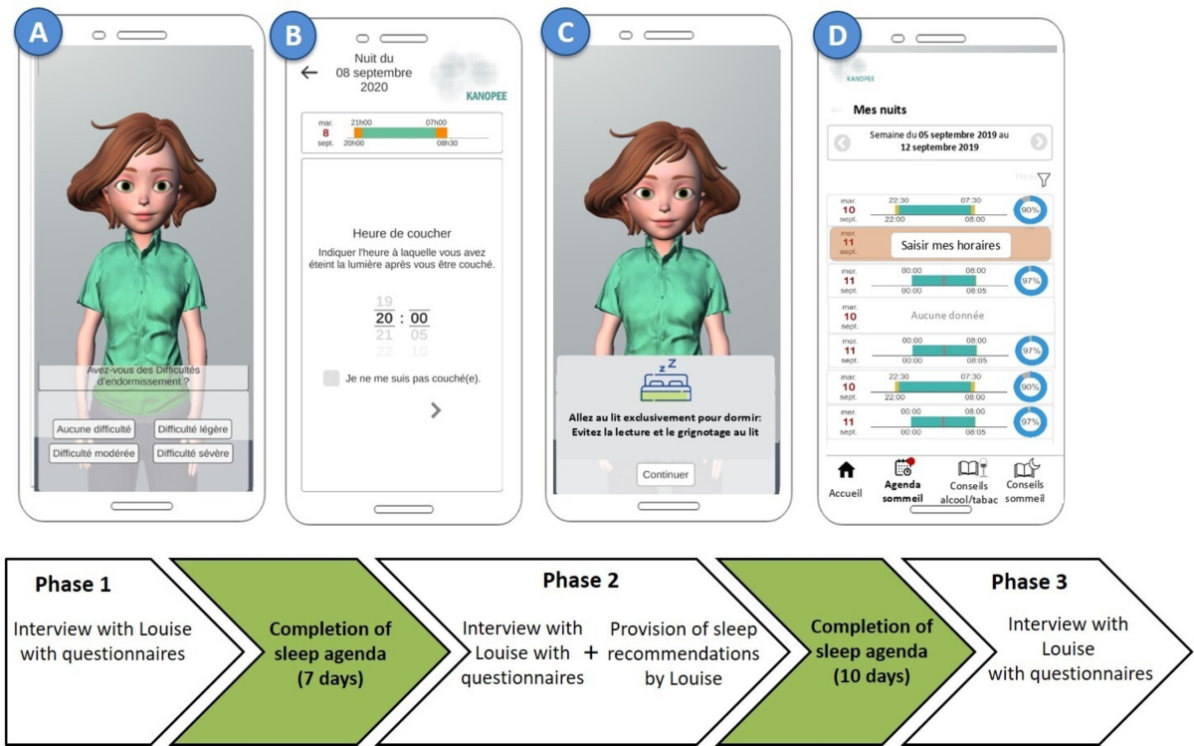
Overview of phases of the KANOPEE digital intervention. (A) screenshot of Louise administering the Insomnia Severity Index; (B) screenshot of the sleep agenda or diary; (C) screenshot of a sleep recommendation given by Louise during phase 2; (D) screenshot of visual feedback provided by the app on the completion of each day in the sleep agenda or diary.

KANOPEE is a smartphone app available on Google Play Store and Apple Store in France that was launched during the peak of the COVID-19 pandemic in 2020. The app includes a VA able to engage in face-to-face dialogue through verbal and nonverbal behavior. Figure 1 summarizes the flow of the intervention. As shown, it included 3 main phases. During the screening interview (phase 1), the VA, named Louise, introduced herself and administered, among other measures, the Insomnia Severity Index (ISI), as the main outcome of the intervention [33]. Then visual feedback was provided to users regarding the severity of their insomnia symptoms, with the help of a colored line featuring the traffic-light colors (green, orange, and red), and they were invited to begin a personalized program. After completing this interview, users were asked to complete a sleep diary regarding their previous night. Once this step was completed, another screen informed the users that they needed to complete the same sleep diary for 7 days, every morning, and that, after these 7 days, the VA would re-evaluate their sleep in another interview and would analyze the information provided in their sleep diaries. An icon with the name “Sleep diary” appeared then at the bottom of the screen that the user could access every day to answer the sleep-related questions (ie, what time did you go to bed last night?). Daily reminders were not provided. After users completed the sleep diary for 7 days, Louise conducted another interview (phase 2) in which users learned about their sleep patterns from the previous week and completed the ISI for the second time, along with other questionnaires. Next, Louise provided sleep recommendations, highlighting the specific ones most useful to the user, based on their sleep diary data and on their answers to the ISI questions. Apart from general good sleep hygiene practices, Louise proposed evidence-based behavioral recommendations that have been shown to improve insomnia symptoms. These instructions were part of the stimulus control component of CBT-I [15]. The goals of stimulus control were (1) to remove the association between the bed or bedroom and wakefulness in order to restore the association of the bed or bedroom with sleep and (2) to establish a consistent wake time. Stimulus control instructions included the following: go to bed only when sleepy, get out of bed when unable to sleep, use the bed or bedroom for sleep (no reading, eating, watching TV, etc, in bed), and wake up at the same time every morning [15]. Next, individuals were asked to complete the sleep diary for 10 more days. After this, they were again interviewed by Louise (phase 3) and completed the ISI, along with other questionnaires. After this final interview, they could choose to continue using the app, if they wished (ie, completing sleep diaries for a longer period). If they considered that their sleep problems were persisting, they were prompted to consult a sleep specialist. Further details about the design of the VA and the design and implementation of the intervention program are described elsewhere [21,22].

### Measures

This study’s main outcome variable, early dropout, was defined as completing phase 1 and not returning to the app. Early dropout was operationalized as a binary outcome: having dropped out versus having completed all the treatment phases.

The ISI scores at phase 1 were used to classify users into 2 groups: users with clinical insomnia and those with subthreshold insomnia. The ISI is a 7-item self-report questionnaire that provides a global measure of perceived insomnia severity during the past month [33]. Scores range from 0 to 28 and can be grouped into the following insomnia severity categories: 0-7 (no insomnia), 8-14 (subthreshold insomnia), 15-21 (insomnia of moderate severity), and 22-28 (severe insomnia). Users were classified as having subthreshold insomnia if their ISI scores ranged between 8 and 14 points and with clinical insomnia if ISI scores were ≥15 points.

Age, sex, and education level were assessed as potential predictors of dropout. Clinical variables included depression, anxiety, and fatigue symptoms. Depression and anxiety symptoms were measured with the Patient Health Questionnaire-4 [34].

Fatigue symptoms were measured with the Fatigue Severity Scale [35]. Sleep characteristics were derived from 1 night of the sleep diary and included the following continuous variables: sleep onset latency, defined as the time taken to fall asleep after turning the lights out; number of awakenings during the night; and sleep efficiency, defined as the ratio of total sleep time to time spent in bed for the night. We also computed a dichotomous variable named short sleep (1=yes, 0=no) if the subject had reported a total sleep time of <6 hours [36].

Additionally, a subset of users completed optional measures related to treatment acceptability and trust in the VA and regarding their level of experience with new technologies. Treatment acceptability was measured with the Acceptability E-Scale (AES) [37]. The AES comprises 6 items and provides a total score as well as 2 subscores regarding usability (ie, the perceived ease of using the app) and satisfaction (ie, pleasure experienced using the app and realizing its value). Items were answered on a 5-point Likert-type scale ranging from 1 (very unsatisfied) to 5 (very satisfied). Trust in the VA was measured with the Embodied Conversational Agent Trust Questionnaire (ETQ) [37]. The ETQ includes 6 items that are answered on a 4-point Likert-type scale ranging from 0 (not at all) to 3 (totally agree). It provides a total score as well as subscores on 2 separate dimensions, named perceived credibility (ie, whether the VA seemed convincing and believable) and perceived benevolence (ie, whether the VA seemed to be of help). Finally, to evaluate users’ level of experience with new technologies, we created a summary score that combined the users’ answers to 3 questions: “Do you use regularly smartphones, tablets, or computers?” with scores ranging from 0 to 3, with higher scores indicating more experience.

### Analyses

A flowchart was first constructed to show the number of users who, once the treatment was initiated (completion of the phase 1 interview), reached each one of our predefined 4 intervention milestones: (1) completing the 7 days of the sleep diary, (2) completing the phase 2 interview, (3) completing 10 days of the sleep diary, and (4) completing the phase 3 interview.

Means and SDs for the continuous variables and percentages and absolute frequencies for the categorical variables were used to describe the study variables. The comparison between the users who completed the intervention (treatment completers) and those who dropped out early (ie, did not return to the app after the phase 1 assessment) was performed by a Student *t* test for continuous variables and a *χ*^*2*^ test for categorical variables.

To identify factors predictive of early dropout in users with subclinical insomnia and in users with clinical insomnia, all factors that showed statistically significant differences between early dropouts and treatment completers in the bivariate analyses were used as predictive variables for multivariable logistic regression analyses. All selected variables were simultaneously inserted into the models (“Enter” method in SPSS Statistics).

Finally, a separate multivariate logistic regression model was tested, including additional variables such as treatment acceptability, trust in the VA, and experience using new technologies, within the subgroup of users having insomnia symptoms (either clinical or subclinical) who completed these optional measures. Results were presented as OR and 95% CIs, with their corresponding *P* values. The level of statistical significance was set as *P*<.05. All analyses were performed with SPSS, version 28 (IBM).

## Results

**Figure 2.**
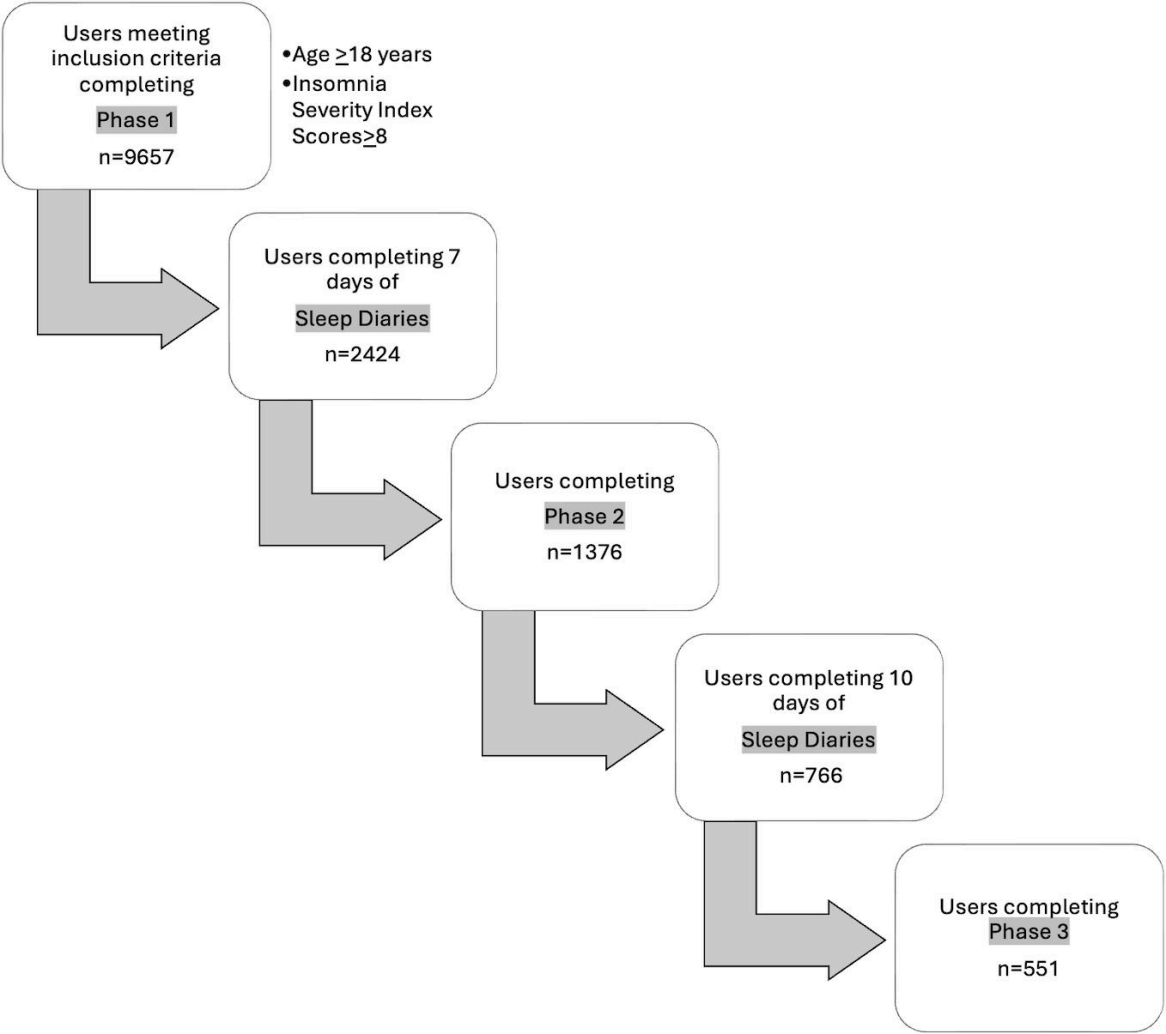
Number of users completing each intervention milestone.

Of the 9657 individuals who downloaded and completed the phase 1 interview during the study period and met the inclusion criteria, a total of 4295 (44.5%) dropped out and did not return to the app to continue filling in the sleep diary for a second day. These users were considered early dropouts. The flowchart displayed in Figure 2 shows the number of users completing each one of the 4 intervention milestones. A total of 551 users reached all the milestones (treatment completers).

### Participants’ Characteristics

We divided users in 2 groups, according to the severity of their ISI scores in phase 1. Within the early dropouts (n=4295), 2166 users had ISI scores within the clinical range (≥15 points) and 2129 users had subclinical ISI scores (8‐14 points). Among treatment completers (n=551), 209 users had subclinical ISI scores and 342 users had ISI scores ≥15 points.

Tables1  2 show the characteristics of users who dropped out and those who completed the intervention, as assessed in the phase 1 screening interview. Within the group of clinical insomnia sufferers (Table 1), users who dropped out were younger, had more severe mental health symptoms, and had a lower education level than treatment completers (all *P*<.01). Regarding the sleep variables measured in one night of the sleep diary, early dropouts also had poorer sleep efficiency and more awakenings during the night. A similar picture emerged when comparing the characteristics of users with subclinical ISI scores (Table 2): those who dropped out were younger and had a lower education level; more severe anxiety and depression symptoms; and poorer sleep diary measures, such as lower sleep efficiency, more nocturnal awakenings, and longer sleep latency. Furthermore, in the early dropouts group, there was a higher percentage of users with self-reported short sleep (all *P*<.05).

**Table 1.** Sociodemographic, clinical, and sleep characteristics of users with clinical insomnia at phase 1.

User characteristics	Early dropouts (n=2166)	Treatment completers (n=342)	*P* value
Age (in years), mean (SD)	47.40 (14.98)	49.91 (12.47)	<.001
PHQ-4^a^ depression scores, mean (SD)	2.94 (1.86)	2.45 (1.83)	<.001
PHQ-4 anxiety scores, mean (SD)	4.10 (1.72)	3.85 (1.79)	.006
FSS^b^ scores, mean (SD)	5.15 (1.16)	5.10 (1.03)	.20
Sleep diary–derived sleep efficiency, mean (SD)	0.57 (0.32)	0.63 (0.26)	<.001
Sleep diary–derived number of awakenings, mean (SD)	2.26 (1.53)	1.90 (1.40)	<.001
Sleep diary–derived sleep latency (in minutes), mean (SD)	51 (75)	55 (65)	.18
Sex, n (%)			.93
Female	1502 (69.3)	238 (69.6)	
Male	664 (30.7)	104 (30.4)	
Education level, n (%)			<.001
Middle school	446 (20.6)	31 (9.1)	
High school	420 (19.4)	50 (14.6)	
Bachelor’s degree	1020 (47.1)	201 (58.8)	
Master’s degree or higher	280 (12.9)	60 (17.5)	
Sleep diary–derived short sleep, n (%)			.40
Yes	1199 (55.3)	181 (52.9)	
No	967 (44.6)	161 (47.1)	

a PHQ-4: Patient Health Questionnaire-4.

b FSS: Fatigue Severity Scale.

**Table 2.** Table 2. Sociodemographic, clinical, and sleep characteristics of users with subclinical insomnia at phase 1.

User characteristics	Early dropouts (n=2129)	Treatment completers (n=209)	*P* value
Age (in years), mean (SD)	47.70 (15.96)	53.50 (12.31)	<.001
PHQ-4^a^ depression scores, mean (SD)	1.89 (1.66)	1.44 (1.37)	<.001
PHQ-4 anxiety scores, mean (SD)	2.84 (1.81)	2.60 (1.79)	.03
FSS^b^ scores, mean (SD)	4.26 (1.24)	4.15 (1.26)	.21
Sleep diary–derived sleep efficiency, mean (SD)	0.72 (0.28)	0.79 (0.20)	<.001
Sleep diary–derived number of awakenings, mean (SD)	1.60 (1.37)	1.34 (1.20)	.002
Sleep diary–derived sleep latency (in minutes), mean (SD)	34 (40)	29 (54)	.04
Sex, n (%)			.15
Female	1277 (60)	136 (65.1)	
Male	852 (40)	73 (34.9)	
Education level, n (%)			.01
Middle school	321 (15.1)	18 (8.6)	
High school	361 (16.9)	27 (12.9)	
Bachelor’s degree	1117 (52.5)	128 (61.2)	
Master’s degree or higher	330 (15.5)	36 (17.2)	
Sleep diary–derived short sleep, n (%)			.006
Yes	709 (33.3)	50 (23.9)	
No	1420 (66.7)	159 (76.1)	

a PHQ-4: Patient Health Questionnaire-4.

b FSS: Fatigue Severity Scale.

### Predictors of Dropping Out

#### Sociodemographic and Clinical Characteristics

The variables showing a statistically significant association with dropping out in the univariate analyses were used as candidates for the multivariable logistic regression analyses. Table 3 shows the ORs (95% CI) and associated *P* values of the 2 multivariable regression analyses performed; one of these analyses was conducted within the sample of users with clinically significant insomnia symptoms at phase 1, and the other analysis was conducted within the sample of users with subclinical insomnia symptoms at phase 1.

**Table 3.** Adjusted ORs of dropping out according to insomnia severity status. Binary dependent variable: early dropout=1, treatment completion=0.

	Multivariable logistic regression analysis 1:Users with clinical insomnia (n=2508)		Multivariable logistic regression analysis 2:Users with subclinical insomnia (n=2338)	
Variable	OR (95% CI)	*P* value	OR (95% CI)	*P* value
Age (in years)	0.98 (0.97‐0.99)	<.001	0.97 (0.96‐0.98)	<.001
Educational level				
Middle school^a^	1		1	
High school	0.56 (0.35‐0.90)	.02	0.63 (0.33‐1.18)	.14
Bachelor’s degree	0.35 (0.23‐0.52)	<.001	0.38 (0.22‐0.65)	<.001
Master’s degree or higher	0.35 (0.22‐0.55)	<.001	0.46 (0.25‐0.84)	.01
PHQ-4^b^ depression scores	1.12 (1.04‐1.21)	.004	1.21 (1.11‐1.36)	.001
PHQ-4 anxiety scores	1.00 (0.93‐1.1)	.94	0.95 (0.86‐1.04)	.28
Sleep diary–derived sleep efficiency	0.64 (0.42‐0.96)	.03	0.37 (0.14‐0.95)	.04
Sleep diary–derived number of awakenings	1.13 (1.04‐1.23)	.003	1.16 (1.03‐1.30)	.015
Sleep diary–derived sleep latency (in minutes)	N/A		1.00 (1.00‐1.00)	.79
Sleep diary–derived short sleep	N/A			
Yes			1.13 (0.72‐1.79)	.59
No^a^			1	

a Reference category.

b PHQ-4: Patient Health Questionnaire-4.

Within the subgroup of users with clinical insomnia, the analyses showed that sociodemographic variables were related to dropout; as age increased, the odds of dropping out decreased. Furthermore, individuals with a higher educational level than middle school had decreased odds of dropping out. More severe depression symptoms and poorer sleep (lower sleep efficiency and a higher number of awakenings during the night) increased the odds of dropping out. As shown in Table 3, a similar set of predictors emerged in the multivariable logistic regression analysis within the group of users with subclinical insomnia symptoms.

#### Perceptions of the App’s Features and Experience Using New Technologies

A total of 1031 users who met the inclusion criteria completed the optional questionnaires regarding their acceptability of the treatment, their trust in the VA, and their experience using new technologies. Compared to treatment completers (n=184), users dropping out (n=847) had lower mean scores on the AES (mean 26.13, SD 4.16 vs mean 27.14, SD 3.40, respectively, *P*<.001) and on the ETQ (mean 18.90, SD 3.26 vs mean 19.82, SD 2.67, respectively, *P*<.001) and reported having less experience using new technologies (mean 1.85, SD 0.75 vs mean 2.07, SD 0.68, *P*<.001). Table 4 shows the ORs of the multivariable logistic regression analysis including these 3 additional variables besides the statistically significant variables included in Table 3. Older age, having a bachelor’s or master’s degree, and more trust in the VA (ie, higher ETQ scores) reduced the odds of dropping out, whereas higher depression scores increased the odds of dropping out (all *P*<.01).

**Table 4.** Adjusted ORs of dropping out (n=1031). Binary dependent variable: early dropout=1, treatment completion=0.

Variable	OR (95% CI)	*P* value
Age (in years)	0.98 (0.97‐0.99)	<.001
Educational level		
Middle school^a^	1	
High school	0.57 (0.30‐1.10)	.09
Bachelor’s degree	0.28 (0.17‐0.49)	<.001
Master’s degree or higher	0.31 (0.16‐0.58)	<.001
PHQ-4^b^ depression scores	1.20 (1.09‐1.32)	<.001
Sleep diary–derived sleep efficiency	0.63 (0.33‐1.18)	.15
Sleep diary–derived number of awakenings	1.12 (0.99‐1.27)	.07
AES^c^ total scores	0.96 (0.91‐1.02)	.20
ETQ^d^ total scores	0.91 (0.85‐0.97)	.004
Experience using new technologies	0.80 (0.64‐1.02)	.07

a Reference category.

b PHQ-4: Patient Health Questionnaire-4.

c AES: Acceptability E-Scale.

d ETQ: Embodied Conversational Agent Trust Questionnaire.

## Discussion

The use of fully automated or unguided apps has the potential to increase access to care in a scalable manner. However, in real-word settings, the impact of these digital interventions is limited by their success to engage the users in the therapeutic activities they propose. This study uses real-world data from individuals downloading and completing the first phase of a fully automated smartphone app intervention named KANOPEE, designed to monitor and manage insomnia symptoms using components of CBT-I.

We found that only a small portion of users completed all the intervention phases during the period assessed. The percentage of individuals completing this intervention was 5.7% (551/9657), suggesting low engagement. This percentage is in line with figures shown in other studies examining mental health app usage in extensive real-world settings, with most users downloading the corresponding app but not using it regularly [38,39].

Our results show that sociodemographic characteristics such as being older and having a higher educational level reduced the odds of early dropout, regardless of the severity of the target condition, insomnia. More severe mental health symptoms, such as depression, increased the odds of early dropout. In addition, users were less likely to drop out if they expressed more positive beliefs regarding the benevolence and credibility of the VA that was included in the app.

A recent systematic review on attrition in conversational agent–delivered mental health interventions reports that demographic-related factors do not seem to be associated with dropout [40]. In this review, there is only 1 study that shows that participants dropping out were significantly younger than those who completed the whole intervention [41], as we found herein. The fact that the younger users were more likely to drop out may simply reflect their differential pattern of use of technologies when compared to older individuals. Younger individuals usually have more experience perusing and using digital apps, so they may be more likely to use and quickly discard them, as they are more capable of seeking alternative options.

For some, but not all, of the studies included in the above-cited systematic review, more severe mental health–related baseline symptoms were associated with dropout, such as anxiety, gambling pathology, fear of food, etc, as we found herein. Our findings also dovetail with studies on premature termination of face-to-face CBT-I. In the study by Ong et al [42], patients with shorter total sleep time and more severe depression symptoms were at significantly greater risk of early dropout. Likewise, in the study by Yeung et al [43] exploring predictors of dropout from internet-based self-help CBT-I, more severe depression symptoms predicted a higher risk of treatment noncompletion. These results suggest that users with a greater severity of depression may lose their motivation to follow the intervention more easily and find it more difficult to make the behavioral changes necessary for improving their sleep [44].

An important finding of this study concerns perceptions of VAs and their impact on dropout. Mental health interventions delivered by VAs, conversational agents, or chatbots pretend to simulate the interaction between a mental health expert and the user. Recent research has highlighted that individuals can indeed develop an affective bond with chatbots [45]. Our results support the contention that a VA can provide relevant user stimulation that will eventually pay out in terms of user engagement with a fully automated intervention [46]. It remains to be determined which features of the VA’s interactions may have driven users’ perceptions of its credibility and benevolence. These perceptions represent a promising and potentially modifiable construct that can be targeted to enhance engagement with fully automated treatment [47]. Qualitative studies with end point users should provide further knowledge about specific aspects related to VA-related credibility.

This study has some strengths. First, there is its large sample size. Second, it considered the timing of dropout (ie, after individuals had completed the first treatment phase, provided clinical data, and sufficiently interacted with the app to gain a first impression of it) and explored sociodemographic and clinical factors and users’ perceptions of the treatment associated with dropout at this specific stage. Until now, few studies have examined dropout at different stages of participation [31,42]. Arguably, dropout at other stages may have other determinants. For example, dropout at a later stage may indicate (1) that a user’s symptoms have already improved and that they do not require full exposure to the intervention, (2) that they have developed complications with treatment, or simply (3) that they are dissatisfied with the treatment.

The main limitation of this study is that we only had a reduced number of baseline and sociodemographic variables from the users. Furthermore, the data analyzed in this study are self-reported data. Apart from the bias commonly associated with self-reported data in medical and health research contexts, the fact that the information was provided to a mobile phone app, without any support from a researcher or therapist, may compromise its accuracy to a greater extent.

Our findings should be viewed only as preliminary data for identifying real-world users at risk of early dropout from a fully automated digital intervention for insomnia including a VA. Therefore, these findings are specific to this intervention, so the results cannot be generalized. Future research could assess whether the predictors found herein are valuable for predicting dropout in other fully automated digital interventions targeting insomnia and in different contexts and populations. Information on the reason for dropout was also not collected in this study. Future research should consider interviewing users to obtain a more detailed understanding of the reasons leading to dropout.

Finally, this study has implications for developing a randomized trial that offers an adapted, augmented intervention for individuals who are more likely to drop out.

## Abbreviations

AES: Acceptability E-Scale
AOR: adjusted odds ratio
CBT-I: cognitive behavioral therapy for insomnia
ETQ: Embodied Conversational Agent Trust Questionnaire
ISI: Insomnia Severity Index
VA: virtual agent
